# Prediction of Microsatellite Instability in Colorectal Cancer Using Two Internally Validated Radiomic Models

**DOI:** 10.3390/tomography11110126

**Published:** 2025-11-13

**Authors:** Antonio Galluzzo, Ginevra Danti, Linda Calistri, Diletta Cozzi, Daniele Lavacchi, Daniele Rossini, Lorenzo Antonuzzo, Sebastiano Paolucci, Francesca Castiglione, Luca Messerini, Fabio Cianchi, Vittorio Miele

**Affiliations:** 1 Department of Radiology, Careggi University Hospital, Largo Brambilla 3, 50134 Florence, Italyginevra.danti@gmail.com (G.D.); linda.calistri@unifi.it (L.C.); dilettacozzi@gmail.com (D.C.); vittorio.miele@unifi.it (V.M.); 2 Clinical Oncology Unit, Careggi University Hospital, Largo Brambilla 3, 50134 Florence, Italy; daniele.lavacchi@yahoo.it (D.L.); lorenzo.antonuzzo@unifi.it (L.A.); 3 Department of Experimental and Clinical Medicine, University of Florence, Largo Brambilla 3, 50134 Florence, Italyfabio.cianchi@unifi.it (F.C.); 4 Department of Health Science, University of Florence, Largo Brambilla 3, 50139 Florence, Italy; 5 Medical Oncology Unit, Careggi University Hospital, Largo Brambilla 3, 50139 Florence, Italy; 6 Department of Health Physics, Careggi University Hospital, Largo Brambilla 3, 50134 Florence, Italy; sebastiano.paolucci@studenti.unicam.it; 7 Pathology Unit, Department of Experimental and Clinical Medicine, University of Florence, Largo Brambilla 3, 50134 Florence, Italy; castiglionef@aou-careggi.toscana.it (F.C.); luca.messerini@unifi.it (L.M.); 8 Unit of Digestive Surgery, Careggi University Hospital, Largo Brambilla 3, 50134 Florence, Italy; 9 Department of Experimental and Clinical Biomedical Sciences “Mario Serio”, University of Florence, Largo Brambilla 3, 50134 Florence, Italy

**Keywords:** colorectal neoplasms, microsatellite instability, radiomics, radiomic features, clinical features, precision medicine, overfitting, metrics, IBSI

## Abstract

**Objectives:** To develop two different radiomic models based on preoperative contrast-enhanced computed tomography (PP CT) to predict microsatellite instability (MSI) in patients with colorectal cancer (CRC) before surgery. **Methods:** PP CT scans of 115 CC patients were segmented using 3DSlicer (v5.6.1). Model I included images from three different scanners (GE, Siemens, Philips), while Model II used only one scanner (GE). For Model I, 80 patients were used for training and 35 for internal validation; for Model II, 46 and 24 patients were used, respectively. Data on sex, age, tumour location, and MSI genomic status were collected. A total of 107 radiomic features (RFs) were extracted, and 30 and 35 RFs were identified as relevant for Models I and II, respectively, using the *t*-test or Mann–Whitney test (*p* < 0.05). The most robust RFs were selected using the LASSO regression method. Both models were internally validated. **Results:** Model I, based on 2 RFs and 1 clinical feature (LOCATION) achieved an AUC of 0.76 (95% CI: 0.65–0.87) in the training cohort and 0.74 (95% CI: 0.56–0.92) in the validation cohort. Model II, based on 3 RFs, achieved an AUC of 0.85 (95% CI: 0.73–0.96) in the training cohort and 0.72 (95% CI: 0.50–0.94) in the validation cohort. **Conclusions:** Both radiomic models showed good performance in distinguishing between MSI and non-MSI tumours, potentially reducing the need for invasive histological testing and improving treatment timing. Despite achieving a higher AUC, Model II showed signs of overfitting when compared to Model I, which incorporated two RFs and one clinical feature (LOCATION). Radiomics may function as a non-invasive preoperative screening tool to inform decisions regarding MSI testing and treatment. Building radiomic models on larger, more diverse datasets is preferable to enhance generalizability and reduce overfitting.

## Simple Summary

Microsatellite instability in colorectal cancer plays a crucial role in guiding clinical management and treatment choices. This study investigates the potential of radiomics, an advanced quantitative imaging analysis technique, to detect this genetic feature. Two models developed with different datasets were compared, revealing key statistical differences. Radiomics could help advance personalized medicine by offering faster and more accurate insights, ultimately saving valuable time for patients.

## 1. Introduction

Colorectal cancer (CRC) is the third most diagnosed cancer and the fourth leading cause of cancer-related deaths. Only 5% are related to known hereditary conditions such as Lynch syndrome and familiar adenomatous polyposis. The transition from adenoma to carcinoma occurs over 10 to 15 years, and screening programs include fecal immunochemical test (FIT) and guaiac-based fecal occult blood test (gFOBT); flexible sigmoidoscopy (FS); colonoscopy (CC); and computed tomography colonography (CTC) [1]. There are disparities regarding age, incidence, mortality rates, and time trends across different countries; in particular, more young patients in rich countries develop CRC, reflecting differences in exposure to risk factors, including sedentary lifestyle and several other environmental factors [2]. CTC is probably more accurate than colonoscopy in identifying the exact location of colon tumours, as demonstrated by the study from Offermans et al. [3] where CTC has a lower localization error compared to CC in identifying the correct segment of the CRC, especially in left-sided tumours. This is important because surgical time is longer for right-sided CRC as a consequence of a more complex lymph node dissection, larger tumour size, and the more frequent cases of microsatellite instability (MSI), often associated with greater lymph node invasion [4]. Some studies, moreover, underline how a mobilization of the splenic flexure during left-sided surgery carries a higher risk of complications, especially in female patients and in cases with larger tumours or higher BMI [5]. The CRC stage determines how it will be treated: in stage III it has penetrated the adjacent lymph nodes but has not migrated to the other body parts, and partial colectomy with involvement of adjoining lymph nodes followed by chemotherapy is the standard treatment regimen. Chemotherapy (FOLFOX, XELOX, 5-FU, or MMC) is indicated for advanced tumours defined as T3 tumours with >5 mm invasion (T3cd), or T4 tumours with positive lymph nodes [6]. CT is highly sensitive in detecting wall involvement, and in distinguishing T1–T3ab from T3cd or T4 lesion and a tumour invasion >5 mm [7]. For an accurate assessment of advanced-stage CRC, a combined approach using magnetic resonance images (MRI) and computed tomography (CT) is recommended. In particular, a study comparing CT and 3T MRI for staging the advanced stage of CRC showed that MRI provided superior accuracy [8,9,10]. Molecular profiling of CRC is crucial nowadays and clearly demonstrates that CRCs are not all the same. One of the most significant distinctions lies in CRCs with MSI (MSIc), which account for approximately 15% of all cases. These tumours are more frequently located in the right colon and tend to show poor response to conventional chemotherapy, limiting the effectiveness of standard neoadjuvant treatment strategies. The literature has amply shown that in metastatic patients with MSI-high status (MSI-H), the first-line treatment should be chemo-free. Indeed, MSI-H is a predictive factor for response to immunotherapy, which, compared with chemotherapy, has demonstrated a doubling of progression-free survival (PFS) and a 5-year overall survival rate exceeding 50% [11,12,13]. Nevertheless, they are associated with a better overall prognosis due to their favourable response to immune checkpoint inhibitors: in patients with localized MSI-H CRC, immunotherapy is associated with an exceptionally high rate of both clinical and pathological complete responses. In these patients, in addition to the survival benefits, the possibility of organ preservation is clinically relevant [14]. Moreover, MSIc are often subject to lymph node overstaging, due to the stronger inflammatory response observed in these tumours [15,16]. Given these characteristics, early identification of MSIc could significantly influence therapeutic decision-making and ensure the best treatment for this patient setting, which may potentially be cured with immunotherapy alone. However, the current diagnostic approach typically involves a preoperative biopsy during CC, which has several disadvantages: (1) the necessity of specialized personnel (both medical and nursing), resulting in increased costs and time; (2) the necessity of histopathological assessment based on PCR, immunohistochemistry, or genetic testing, which may also be limited by inadequate or insufficient tissue samples; (3) in up to 15–30% of patients who present to the Emergency Department (ED), this diagnostic step could have been avoided [17]; and (4) the invasive nature of the biopsy may lead to complications, including bleeding or bowel perforation [18]. In the era of artificial intelligence (AI), a future with less invasive diagnostic tools capable of analysing tumour biology preoperatively may not be far off. AI, particularly through radiomic analysis, enables the extraction of quantitative features from radiological images. Radiomics is a field that aims to quantify signal intensities and pixel interrelationships, converting medical images into high-dimensional data to uncover non-intuitive properties of the imaging phenotype and the tumour microenvironment [19]. Several recent studies have shown that differences in imaging equipment, different machines, inadequate patient samples, and the lack of external validation significantly affect the reliability of radiomic models, rendering many studies inconclusive or self-limiting [20,21].

In this study, we have compared two radiomic models, the first based on a larger cohort of patients scanned with different machines, and the second on a smaller group of patients with images obtained exclusively with GE scanners. The aim was not only to identify radiomic features (RFs) potentially associated with microsatellite instability, but also to select the better model. We hypothesised that a CT-based radiomic model could non-invasively predict MSI status in CRC and that a single-scanner model would outperform a multi-scanner model in overall performance.

## 2. Methods

### 2.1. Study Population

**Figure 1:**
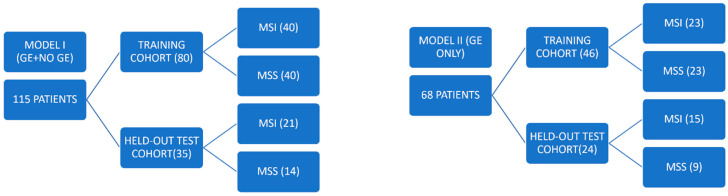
Flowchart of the study population.

This is a retrospective analysis of 138 patients who were referred to our Hospital (Radiology Department of Careggi University Hospital, Florence, Italy) for preoperative CT imaging from August 2016 to November 2024, and who later underwent CRC resection. Patients underwent immunohistochemical analysis either before or after the staging CT scan to assess MSI status. In particular, the preoperative portal venous phase computed tomography (PP CT) scan of the abdomen was included. Patients with suboptimal image quality for segmentation, those who had already started therapy at the time of staging CT, or those whose staging CT was a colonography with oral contrast (where the contrast-filled lumen could interfere with radiomic analysis) were excluded. A total of 115 patients were included in the study (Figure 1). Inclusion criteria were: (1) diagnosis of colon cancer; (2) availability of a preoperative PP CT scan of the abdomen archived in our Picture Archiving and Communication System (PACS); and (3) no treatment initiated before the CT scan. Exclusion criteria were: (1) pediatric age (<18 years); (2) unavailability of preoperative PP CT scan; (3) unavailability of MSI status; (4) bad quality imaging (considered as unreadable or affected by artifacts); and (5) presence of prior biopsies, synchronous or metachronous tumors, metastases, or prior radiotherapy. All patients were equally divided according to genomic status into Microsatellite stability (MSS) patients and MSI patients. Two models were built. In the first one (115 patients, “model I”) no distinction between CT scans (GE, Philips, and Siemens) were made in order to maximize the available sample size; in the second one (70 patients, “model II”) only GE images were considered. In accordance with prior radiomics studies [22], both study cohorts were randomly split into a training cohort (model I: 80 patients; model II: 46 patients) and a held-out test cohort (model I: 35 patients; model II: 24 patients). Each cohort was divided into two groups based on MSI status: Group 0 (MSI) and Group 1 (MSS). For each patient, we collected data on sex, age at the time of diagnosis, and tumour localization within the colon. MSI status was already validated for all patients through genomic analysis. The study was conducted in accordance with the Declaration of Helsinki, and approved by the Ethics Committee of Careggi University Hospital (protocol code 13261_OSS).

### 2.2. Imaging Acquisition

**Figure 2:**
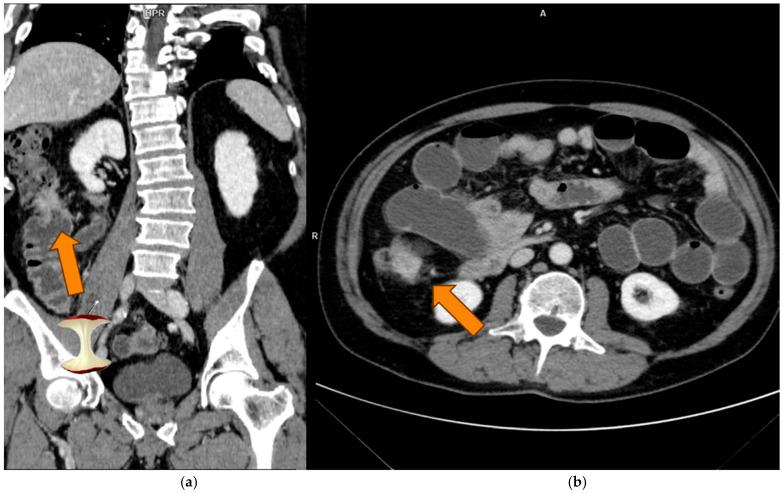
The image shows PP scans of a MSIc of the ascending colon, presenting the classic “apple-core” appearance. Coronal (**a**) and axial (**b**) views are provided.

**Figure 3:**
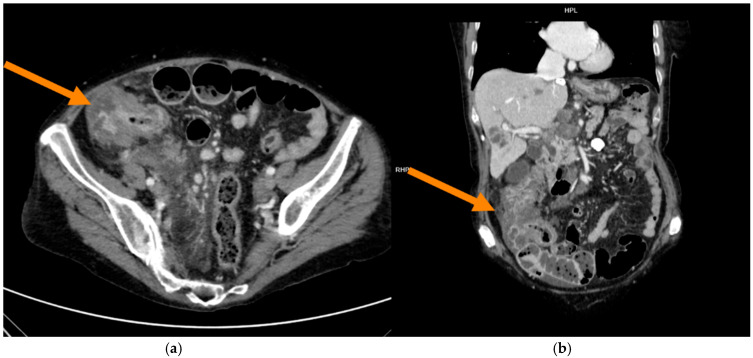
In this case, images demonstrate PP of a MSIc in a more advanced stage, with hepatic metastases. Coronal (**a**) and axial (**b**) views are provided.

CT scans were performed at our centre using a variety of CT manufacturers, including GE, Philips, and Siemens, with models ranging from 16 to 128 slices. All acquisitions performed using different scanners were included in the model I in order to maximize the available sample size; in the model II only GE scans were considered in order to reduce the machine variability. Images were reconstructed with a slice thickness varying between 1 and 3 mm. The mean acquisition parameters were 120 kVp (range: 100–140 kVp). Various contrast media molecules (Iopamiro 400 mg/mL; Iopromide 370 mg/dL; Iobitridolo 350 mg/dL) were used across different studies, with iodine concentrations ranging from 350 to 400 mg/mL at variable dosages. Contrast media were administered intravenously through the antecubital vein at a flow rate of 3.0–3.5 mL/s, and the subsequent saline solution (50 mL) was injected. The bolus-tracking method was the choice for the multiphase CT scan acquisition by setting a 100 HU-threshold region of interest at the abdominal aorta. PP was the most frequently performed type of examination in the preoperatory examination and the most commonly used in the literature for the evaluation of CRC [22]. For this reason it was chosen for the next segmentation phase (Figure 2 and Figure 3).

### 2.3. Lesion Segmentation

**Figure 4:**
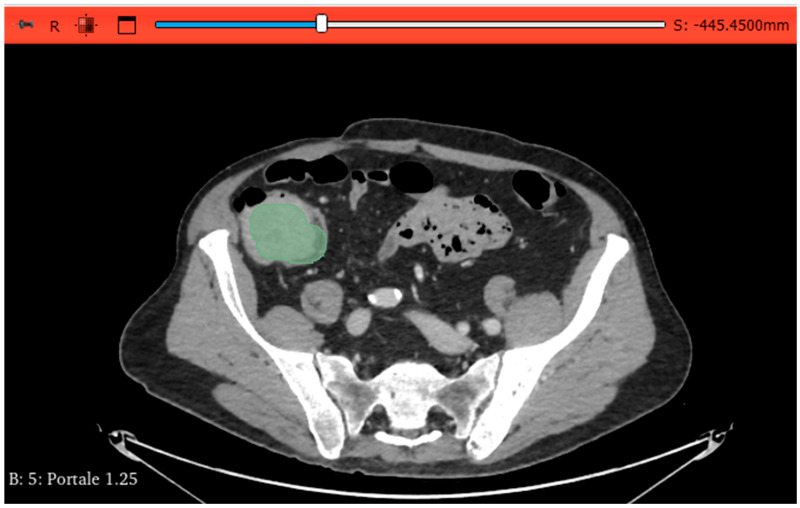
The image-processing software 3D Slicer was used to manually outline ROIs along the lesion margins on all the slices containing the tumour CT venous phase. ROI: region of interest.

All CRC segmentations were afforded by two abdominal radiologists with 4 years of experience, who independently manually performed a volumetric segmentation of the tumour region using 3D Slicer software, version 5.6.1 (open-source software; https://www.slicer.org/, accessed on 22 August 2025) (Figure 4). No intraclass correlation coefficient (ICC) for interobserver repeatability was applied. Air bubbles, the surrounding pericolic fat, and healthy large bowel wall was excluded in the segmentation, which was reviewed and edited by a third, more skilled body imaging radiologist. Radiologists were blinded to MSI status.

### 2.4. Radiomics Feature Extraction and Selection

The RF extraction was made on all slices of the PP where the CRC appeared. The region of interest (ROI) was defined using the SlicerRadiomics extension implemented in the 3D Slicer software, and 107 radiomic features were extracted through the 3D Slicer Radiomics extension (Pyradiomics (v2.1.2)). The extracted RFs were then subdivided into three main classes: “shape-based”, first order statistics, and second order statistics. “Shape-based” features describe the shape and size of the ROI; “first order (I-order) statistics” are calculated from the intensity histogram of the image; and “second-order (II-order) statistics” provide the spatial arrangement correlation of the intensity values within the ROI [23]. In order to utilize RFs as predictors in the machine learning (ML) model, and to avoid overfitting, only certain features were selected to create the ML model. The dataset was already divided into a training subset and a testing subset; in particular, in the first model, the study cohort was randomly split into a training cohort (80 patients) and a held-out test cohort of validation (35 patients). Each cohort was divided into two groups based on MSI status: Group 0 (MSI) and Group 2 (MSS). In the second model the study cohort was randomly split into a training cohort (46 patients) and a held-out test cohort of validation (24 patients). Each cohort was divided into two groups based on MSI status: Group 0 (MSI) and Group 1 (MSS).

Through statistical analysis, significant features in the training cohort were identified, and the radiomic model was then validated in the held-out test cohort according to the Image Biomarker Standardisation Initiative (IBSI) and Metrics guidelines, both in model I (Table 1) and in model II (Table 2) [24,25].

### 2.5. Statistical Analysis

For both models in the training cohort, a total of 107 RFs were extracted. Neither rescaling nor specific image filters were applied. However, for gray-level discretization (to extract second-order features), a fixed bin width (FBW) approach was used with a bin width = 4. The Shapiro–Wilk test and Levene’s test were, respectively, used to assess the normality of data distribution within each group and the homogeneity of variances between groups based on the resulting *p*-values. Student’s *t*-test or the Mann–Whitney U test was then applied to evaluate statistically significant differences in feature distributions between Groups 0 and 1. A radiomic feature (RF) was considered “statistically significant” if the *p*-value was <0.05. RFs with *p*-values below the Bonferroni-corrected threshold of 0.05/107 = 0.000467 were considered “statistically significant after Bonferroni correction”, which accounted for multiple comparisons across the 107 RFs. The least absolute shrinkage and selection operator (LASSO) regression method, which allows for both feature regularization and selection, was then applied within cross validation folds to select the most relevant features in order to build the model. Along with the radiomic model, Receiver Operating Characteristic (ROC) curves and Area Under the Curve (AUC) values were generated, and a 95% confidence interval (CI) was reported. A five-fold cross-validation via minimum criteria was used to select the optimal penalty term (λ). Specifically, 100 λ values were explored over a logarithmic grid. For each λ, the mean error across the five test folds was computed. The optimal λ, corresponding to the minimum mean error, was subsequently selected for model development. This trained model was then validated with the internal validation cohort. The Youden Index was used to identify the optimal decision threshold for classifying patients into Group 0 or Group 1 in both models. In order to avoid high performance variance and to estimate the 95% confidence intervals, a bootstrap procedure (B = 2000) was applied.

## 3. Results

A total of 128 patients with CRC and known MSI status who underwent PP CT between August 2016 and November 2024 were part of this study. Some patients did not meet the inclusion criteria and were excluded from the analysis (23 patients). A final number of 115 patients were included in the study. Demographic characteristics of the patients are reported in Table 3.

**Figure 5:**
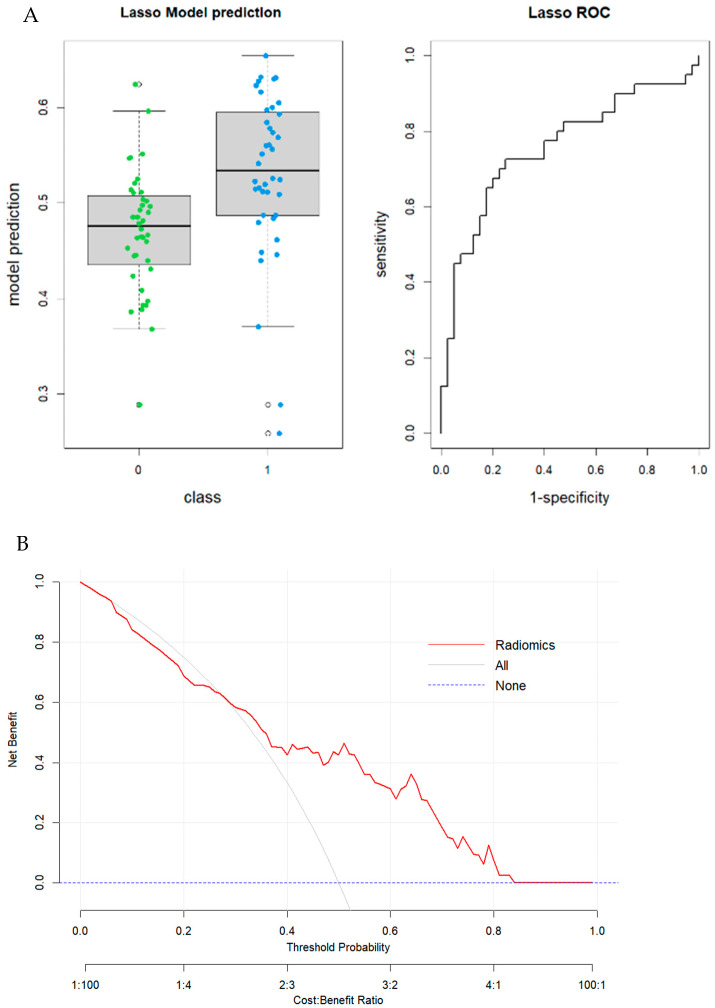
Model I. (**A**) Boxplot of performance in prediction models (left) and ROC curves (with AUC) in the training cohort; Prediction Logistic Regression models were obtained with LASSO algorithm (AUC = 0.76 (95% CI: 0.65–0.87; (DeLong)). (**B**) Decision curve analysis (DCA) of the training cohort.

**Figure 6:**
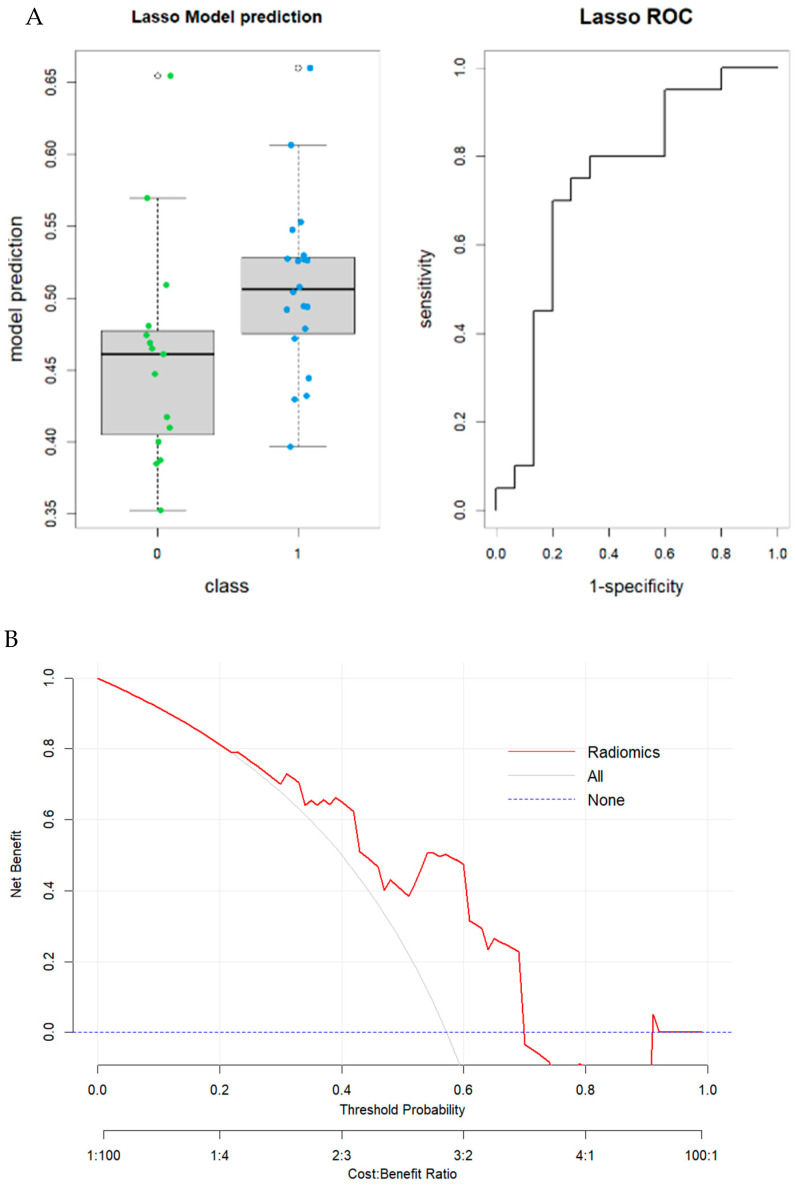
Model I. (**A**) Boxplot of performance in prediction models (left) and ROC curves (with AUC) in the validation cohort; Prediction Logistic Regression models were obtained with LASSO algorithm (AUC = 0.74 (95% CI: 0.56–0.92 (DeLong)). (**B**) Decision curve analysis (DCA) of the validation cohort.

The cohort comprised 63 males and 52 females, with an average age at diagnosis of 66.15 years. Among these, 68 patients underwent scans on a GE CT scanner, and 47 on different models. In model I, which involved 115 patients, 30 features (*p*-value threshold = 0.05) were considered significative using the Mann–Whitney or *t*-test, comprising six shape, three first order, four GLCM, six GLDM, six GLRLM, six GLSZM, and four NGTDM features (Table 2). The best-performing prediction model was generated using the LASSO penalization regression method applied to the significant features, and the following three features were selected: clinical feature “LOCATION”; shape feature shape_SurfaceVolumeRatio; and second-order features “gldm_DependenceEntropy”. The radiomic model achieved a good performance in predicting MSI status in colon cancer with an AUC value of 0.76 (95% CI: 0.65–0.87; DeLong). Boxplots of prediction model performance and ROC curves with AUC values are represented in Figure 5. The Youden index was used to identify the optimal decision threshold that maximizes the sum of sensitivity and specificity. At the maximum Youden index, the corresponding specificity, sensitivity, accuracy and precision were computed. The optimal cutoff value in the first model was 50.6%, yielding a sensitivity of 0.72 (95% CI: [0.58–0.85]); and a specificity of 0.75 (95% CI: [0.61–0.88]), respectively. The results were validated through the internal cohort, in which the radiomic model achieved an AUC value of 0.74 (95% CI: 0.56–0.92; DeLong). Boxplots of prediction model performance and ROC curves with AUC values both in the training and validation dataset are represented in Figure 6. The model achieved a satisfying accuracy (0.63); low sensitivity (0.5) (95% CI: [0.29–0.74]); high specificity (0.8) (95% CI: [0.60–1.00]); and precision (0.77). Regarding the inclusion of clinical data to enhance model performance, only tumour localization improved the model.

**Figure 7:**
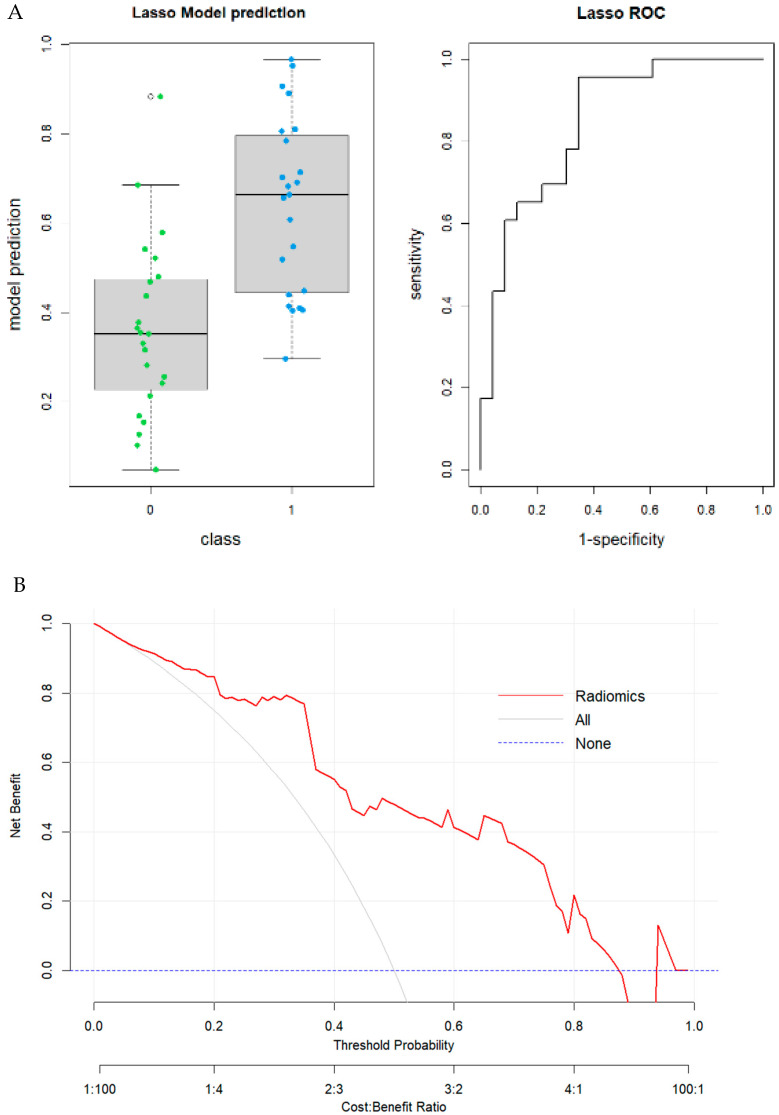
Model II. (**A**) Boxplot of performance in prediction models (**left**) and ROC curves (with AUC) in the training cohort; Prediction Logistic Regression models were obtained with LASSO algorithm (AUC = 0.85 (95% CI: 0.73–0.96; (DeLong)). (**B**) Decision curve analysis (DCA) of the training cohort.

**Figure 8:**
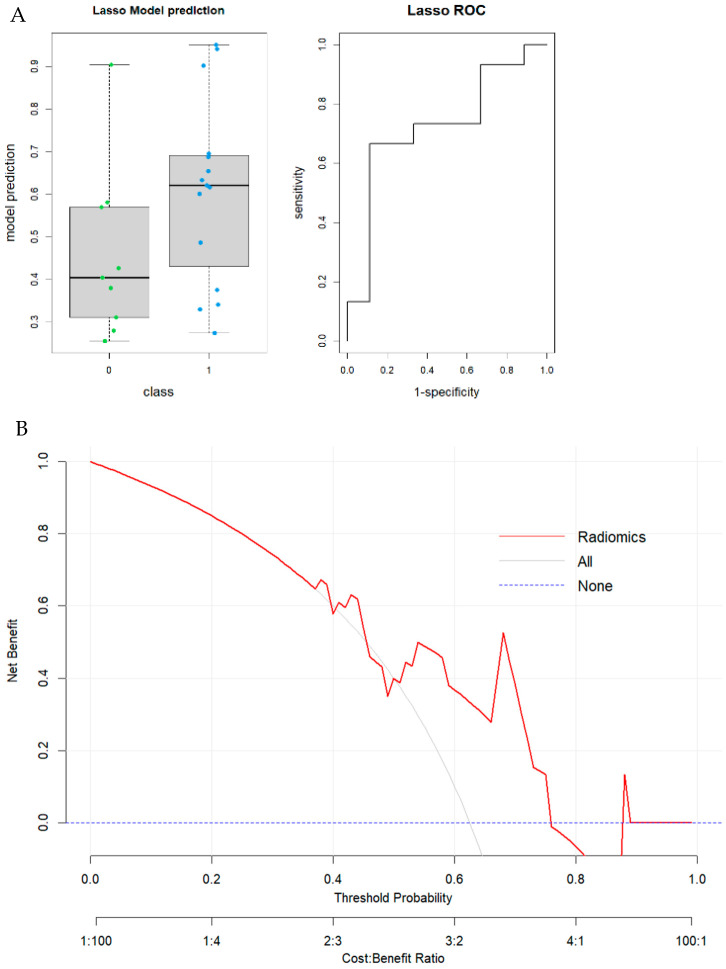
(**A**) Model II. Boxplot of performance in prediction models (**left**) and ROC curves (with AUC) in the training cohort; Prediction Logistic Regression models were obtained with LASSO algorithm (AUC = 0.72 (95% CI: 0.50–0.94; (DeLong)). (**B**) Decision curve analysis (DCA) of the validation cohort.

In model II, 68 patients were involved. A total of 35 features (*p*-value threshold = 0.05) were considered significative using the Mann–Whitney or *t*-test, comprising 11 shape, two first order, four GLDM, six GLRLM, five GLSZM, and two NGTDM features (Table 1). The LASSO regression method selected three RFs: first order “Skewness”; second order “glcm_Autocorrelation”; and “gldm_SmallDependenceLowGrayLevelEmphasis”. The radiomic model achieved a good performance in predicting MSI status in colon cancer with an AUC value of 0.85 (95% CI: 0.73–0.96; DeLong). Boxplots of prediction model performance and ROC curves with AUC values are represented in Figure 7. The Youden Index was used to identify the optimal decision threshold for classifying patients into Group 0 or Group 1. The optimal cutoff value in the second model was 39.1%, yielding a sensitivity of 0.957 (95% CI: [0.85–1.00]) and a specificity of 0.652 (95% CI: [0.44–0.84]), respectively. The results were validated through the internal cohort, in which the radiomic model achieved an AUC value of 0.72 (95% CI: 0.50–0.94; DeLong). Boxplots of prediction model performance and ROC curves with AUC values both in the training and validation dataset are represented in Figure 8. The model achieved a satisfying accuracy (0.63); low sensitivity (0.73) (95% CI: [0.50–0.94]); low specificity (0.44) (CI: [0.13–0.78]); and precision (0.69). To assess model calibration, the Brier score (ranging from 0 = perfect to 1 = worst) was computed as follows: total cohort—training group: Brier score = 0.25 (RMSE = 0.50); total cohort—test group: Brier score = 0.23 (RMSE = 0.48); GE-only cohort—training group: Brier score = 0.17 (RMSE = 0.41); and GE-only cohort—test group: Brier score = 0.22 (RMSE = 0.47). The results are summarized in Table 4.

## 4. Discussion

**Figure 9:**
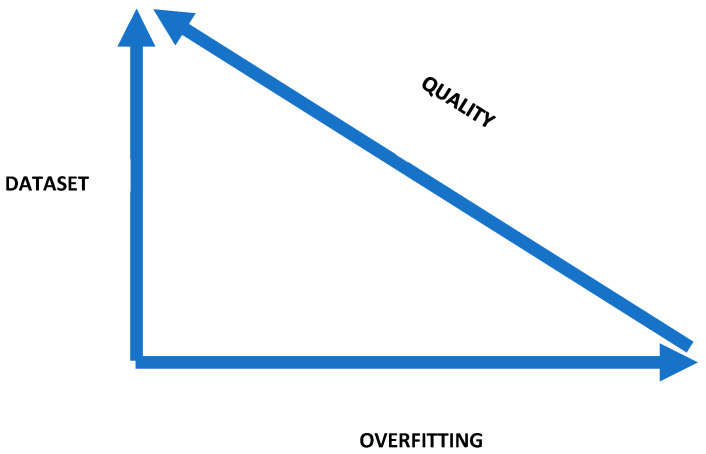
The image illustrates how an insufficiently sized dataset reduces the quality of radiomic studies by increasing overfitting.

There is a growing need for objective, quantitative data that can serve as a tool to overcome challenges in diagnostic accuracy and timing. MSI status is a key prognostic marker in CRC, as it is associated with a lower risk of metastatic spread in early-stage (I–II) disease. However, MSIc tend to respond poorly to standard chemotherapy in advanced stages (III–IV), necessitating the use of targeted therapies such as Pembrolizumab [11,26]. Radiomics aims to advance precision oncology by integrating diagnostic and prognostic imaging biomarkers. These biomarkers can help predict treatment response and monitor disease progression, allowing for more personalized patient management. This quantitative technique has already shown potential in identifying BRAF and RAS mutations (genomic signatures linked to poorer outcomes) potentially avoiding the need for invasive biopsies, which carry risks such as bleeding and patient discomfort [27]. Similarly, the primary objective of this study was therefore to develop a radiomic model able to predict MSI status in CRC noninvasively, reducing the need for tissue sampling and supporting tailored therapies like immunotherapy. This was achieved through volumetric segmentation of primary tumours on preoperative PP CT scans. The secondary aim was to compare two radiomic models: one developed using a larger dataset (Model I) and another with reduced variability in CT scanner types (Model II). All patients underwent surgical resection, and histopathological analysis was performed. Clinical data including age, sex, and tumour localization were considered. Model I, based on a larger dataset, achieved an AUC of 0.76 for predicting MSI in CRC, and maintained good performance in internal validation, yielding an AUC of 0.74. Model II, developed with reduced scanner variability, demonstrated a higher AUC of 0.85 in the primary analysis; however, its performance decreased to an AUC of 0.72 in internal validation. Our results are in line with those of previous studies, some of which reported even higher AUC values [28,29]; however, in our study, we also assessed the differences between the two types of models described above. Statistical analysis revealed an interesting finding: the clinical variable “LOCATION” was selected by LASSO regression method in the analysis of model “I”, but it was not selected in model “II”. A comparison in model II of the two ROC curves using DeLong’s test for independent samples produced Z = 0.43 (two-sided *p* = 0.67), indicating that the observed difference in AUCs (Δ = 0.12) is not statistically significant. No evidence of model overfitting was therefore detected; however, the external validation cohort was small (n = 24) and confidence intervals are wide, which reduces the power to detect modest differences. Measures have been adopted to reduce the impact of overfitting (such as the LASSO regression method and a 5-fold cross-validation via minimum criteria), Specifically, the model complies with the commonly accepted rule of thumb stating that the number of features should be less than one tenth of the total sample size [30]. Overfitting is an excessive reliance of the model on training data, leading to inability to generalise and evaluate other data well. In other words, it is a misleadingly better performance of models built on small datasets compared to those developed on larger cohorts (which also explains the higher AUC of model “II”), leading to an overestimation of model validity [31]. When a radiomic model is built on a very small cohort, the importance of certain RFs tends to be overestimated. This is demonstrated by the fact that in model II, three RFs and no clinical features were selected, despite clinical features being independent of the number of patients. From a ML analysis perspective, a lower number of RFs avoids the potential problem of overfitting, leading to an increase in overall performance [32]. The lack of a large dataset precludes proper internal validation, which in turn may render the study self-referential and increase the risk of overfitting [33]. A potential solution is to use larger training datasets and reduce model complexity (Figure 9). Another important conclusion drawn from this study, which increases the validity of model I, is that the key clinical features (LOCALIZATION) align with what is reported in the literature; that is, MSIc are predominantly located in the right colon. Several radiomic comparative studies evaluate different patient datasets, but few clearly highlight the issue of overfitting between two radiomic models trained on datasets of differing sizes [34]. In this study, we also evaluated the impact of heterogeneous CT scanner usage on model performance. As discussed above, the application of radiomics to CT imaging has been explored in various clinical contexts related to CRC [27]. Its potential in predicting lymph node metastasis [35], as well as gene mutations such as KRAS, NRAS, and BRAF [36,37] has been evaluated. Radiomics has also shown promise in identifying tumours resistant to treatment [38], and in predicting treatment response in colorectal liver metastases [39] and disease-free survival [40]. Furthermore, delta-radiomics, a technique assessing temporal changes in RFs, may offer valuable insights into treatment response and disease prognosis [41,42]. The potential advantages of radiomics are numerous, starting with the development of tailored therapies, avoiding the treatment of patients unlikely to respond to chemotherapy, and enabling more effective monitoring of oncologic patients during follow-up. However, the limitations of radiomics are equally well-recognized, including issues related to standardization and generalizability of results, data quality control, repeatability and reproducibility, database compatibility, and risks of model overfitting. Radiomic studies often differ significantly in their approaches to image segmentation, feature extraction, model construction, and validation strategies, and are frequently affected by unbalanced datasets [43]. In our study, in order to minimize errors and variability, the IBSI and METRICS guidelines were followed [24,25]. However, the lack of external validation remains a limitation, as it would have further strengthened the scientific validity and enhanced the generalizability of the model, given that the predicted outcomes were not externally confirmed.

This study has several other limitations, including its retrospective design, the relatively small sample size, the lack of assessment of inter-observer repeatability, and the absence of follow-up data. Another limitation of the present study concerns scanner and acquisition variability. Although Model I was designed to ensure broader generalizability compared to Model II, neither inter-scanner harmonization nor intensity standardization was ultimately applied. We initially attempted to perform inter-scanner harmonization using the ComBat method; however, this approach resulted in poorer outcomes, increasing scanner dependency compared to the non-harmonized data. For this reason, the method was not adopted, and this variability may have influenced the extracted radiomic features. Moreover, this study lacks a comparison between different models, such as clinical-only and combined models, and this aspect warrants further investigation in future research. More studies should aim to include larger patient cohorts and adopt a prospective design. Key steps toward clinical integration of radiomics include establishing standardized imaging acquisition protocols and conducting multicentre prospective trials. The subsequent stages of clinical translation require robust external and multicentre validation, regulatory approval as medical software, and seamless PACS integration to ensure that radiomics tools function automatically, securely, and in a clinically interpretable way within existing workflows. As described by Chen et al., a radiomic calculator could be embedded within the PACS to automatically extract features from segmented lesions in lung cancer and generate risk scores, which could be added to radiology reports or used in multidisciplinary meetings to support decision-making [44].

## 5. Conclusions

This study confirms the potential of radiomic models based on preoperative CT to non-invasively predict MSI in CRC. Two internally validated models were compared, revealing how dataset size and scanner variability affect performance and risk of overfitting. Model I, built on a larger dataset, showed more stable results and incorporated clinically relevant features, while Model II lacked generalizability. These findings highlight the importance of robust datasets, internal validation, and standardization in radiomic research. Despite limitations such as retrospective design and lack of external validation, this work lays the groundwork for future multicentre prospective studies toward clinical implementation.

## Figures and Tables

**Table 1:** Significant features identified in the training cohort in model I through statistical analysis.

SFs (30)	Test	*p*-Value	Bonferroni *p*-Value	SB	α/µ Differences	α/µ Group 0	α/µ Group 1
shape_LeastAxisLength	TT	0.00614	0.0004673	NO	α_0_ < α_1_	24.8	33.1
shape_MajorAxisLength	MWT	0.00981	0.0004673	NO	µ_0_ < µ_1_	44.7	56.5
shape_Maximum2DDiameterColumn	MWT	0.01855	0.0004673	NO	µ_0_ < µ_1_	45.7	59.9
shape_Maximum2DDiameterRow	MWT	0.03253	0.0004673	NO	µ_0_ < µ_1_	45.3	57.5
shape_Maximum2DDiameterSlice	MWT	0.00873	0.0004673	NO	µ_0_ < µ_1_	46.7	56.4
shape_Maximum3DDiameter	MWT	0.01100	0.0004673	NO	µ_0_ < µ_1_	51.9	69.0
shape_MeshVolume	MWT	0.00508	0.0004673	NO	µ_0_ < µ_1_	10,411	31,212
shape_MinorAxisLength	MWT	0.01956	0.0004673	NO	µ_0_ < µ_1_	34.4	40.1
shape_SurfaceArea	MWT	0.01454	0.0004673	NO	µ_0_ < µ_1_	6489	11,869
shape_SurfaceVolumeRatio	MWT	0.00610	0.0004673	NO	µ_0_ ≥ µ_1_	0.52	0.40
shape_VoxelVolume	MWT	0.00508	0.0004673	NO	µ_0_ < µ_1_	10,429	31,250
firstorder_Energy	MWT	0.01666	0.0004673	NO	µ_0_ < µ_1_	85,191,293	137,981,938
firstorder_TotalEnergy	MWT	0.01339	0.0004673	NO	µ_0_ < µ_1_	85,232,670	184,705,911
gldm_DependenceEntropy	MWT	0.01131	0.0004673	NO	µ_0_ < µ_1_	6.96	7.04
gldm_DependenceNonUniformity	MWT	0.00754	0.0004673	NO	µ_0_ < µ_1_	3473.5	5893.0
gldm_GrayLevelNonUniformity	MWT	0.00358	0.0004673	NO	µ_0_ < µ_1_	672	1382
gldm_SmallDependenceEmphasis	MWT	0.02599	0.0004673	NO	µ_0_ ≥ µ_1_	0.381	0.323
glrlm_GrayLevelNonUniformity	MWT	0.00358	0.0004673	NO	µ_0_ < µ_1_	614.3	1273.8
glrlm_LongRunEmphasis	MWT	0.03763	0.0004673	NO	µ_0_ < µ_1_	1.21	1.24
glrlm_RunLengthNonUniformity	MWT	0.00557	0.0004673	NO	µ_0_ < µ_1_	12,624	23,665
glrlm_RunPercentage	MWT	0.04237	0.0004673	NO	µ_0_ ≥ µ_1_	0.939	0.931
glrlm_RunVariance	MWT	0.03673	0.0004673	NO	µ_0_ < µ_1_	0.071	0.081
glrlm_ShortRunEmphasis	MWT	0.04871	0.0004673	NO	µ_0_ ≥ µ_1_	0.954	0.948
glszm_GrayLevelNonUniformity	MWT	0.00848	0.0004673	NO	µ_0_ < µ_1_	244.8	409.8
glszm_SizeZoneNonUniformity	MWT	0.01622	0.0004673	NO	µ_0_ < µ_1_	3096.3	3829.9
glszm_SizeZoneNonUniformityNormalized	MWT	0.01622	0.0004673	NO	µ_0_ ≥ µ_1_	0.419	0.394
glszm_SmallAreaEmphasis	TT	0.02510	0.0004673	NO	µ_0_ ≥ µ_1_	0.677	0.655
glszm_ZoneEntropy	TT	0.00349	0.0004673	NO	µ_0_ < µ_1_	6.7	6.9
ngtdm_Busyness	MWT	0.04138	0.0004673	NO	µ_0_ < µ_1_	0.445	0.976
ngtdm_Coarseness	MWT	0.00462	0.0004673	NO	µ_0_ ≥ µ_1_	0.00056	0.00031

MWT: Mann–Whitney test; TT: *t*-test; µ_0_: median group 0; µ_1_: median group 1; α_0:_ mean group 0; α_1_ mean group: SFs: significant features; SB: significative for Bonferroni. gldm: Gray Level Dependence Matrix.

**Table 2:** Significant features identified in the training cohort in model II through statistical analysis.

SFs (35)	Test	*p*-Value	Bonferroni *p*-Value	SB	α/µ Differences	α/µ Group 0	α/µ Group 1
shape_MajorAxisLength	MWT	0.03403	0.0004673	NO	µ_0_ < µ_1_	43.22	51.38
shape_Maximum2DDiameterSlice	TT	0.01502	0.0004673	NO	α_0_ < α_1_	48.10	60.19
shape_Maximum3DDiameter	MWT	0.02130	0.0004673	NO	µ_0_ < µ_1_	51.97	69.90
shape_MeshVolume	MWT	0.04990	0.0004673	NO	µ_0_ < µ_1_	10,688	35,614
shape_SurfaceVolumeRatio	MWT	0.04732	0.0004673	NO	µ_0_ ≥ µ_1_	0.607	0.410
shape_VoxelVolume	MWT	0.04732	0.0004673	NO	µ_0_ < µ_1_	10,736	35,670
firstorder_Kurtosis	MWT	0.00861	0.0004673	NO	µ_0_ ≥ µ_1_	6.08	3.16
firstorder_Minimum	MWT	0.02319	0.0004673	NO	µ_0_ < µ_1_	−127	−4
firstorder_Skewness	MWT	0.00804	0.0004673	NO	µ_0_ < µ_1_	−0.908	−0.368
glcm_Autocorrelation	MWT	0.02866	0.0004673	NO	µ_0_ ≥ µ_1_	2662	499
glcm_Idn	MWT	0.04990	0.0004673	NO	µ_0_ ≥ µ_1_	0.944	0.919
glcm_JointAverage	MWT	0.02866	0.0004673	NO	µ_0_ ≥ µ_1_	51.25	21.83
glcm_SumAverage	MWT	0.02866	0.0004673	NO	µ_0_ ≥ µ_1_	102.5	43.7
gldm_HighGrayLevelEmphasis	MWT	0.02549	0.0004673	NO	µ_0_ ≥ µ_1_	2650	502
gldm_LargeDependenceHighGrayLevelEmphasis	MWT	0.00922	0.0004673	NO	µ_0_ ≥ µ_1_	25,035	5201
gldm_LargeDependenceLowGrayLevelEmphasis	MWT	0.03215	0.0004673	NO	µ_0_ < µ_1_	0.0039	0.0333
gldm_LowGrayLevelEmphasis	MWT	0.02130	0.0004673	NO	µ_0_ < µ_1_	0.0009	0.0062
gldm_SmallDependenceHighGrayLevelEmphasis	MWT	0.04732	0.0004673	NO	µ_0_ ≥ µ_1_	866	161
gldm_SmallDependenceLowGrayLevelEmphasis	MWT	0.03215	0.0004673	NO	µ_0_ < µ_1_	0.00061	0.00330
glrlm_HighGrayLevelRunEmphasis	MWT	0.02549	0.0004673	NO	µ_0_ ≥ µ_1_	2649	501
glrlm_LongRunHighGrayLevelEmphasis	MWT	0.02130	0.0004673	NO	µ_0_ ≥ µ_1_	3051	618
glrlm_LongRunLowGrayLevelEmphasis	MWT	0.01373	0.0004673	NO	µ_0_ < µ_1_	0.00094	0.0073
glrlm_LowGrayLevelRunEmphasis	MWT	0.02004	0.0004673	NO	µ_0_ < µ_1_	0.00089	0.0065
glrlm_ShortRunHighGrayLevelEmphasis	MWT	0.02704	0.0004673	NO	µ_0_ ≥ µ_1_	2514	471
glrlm_ShortRunLowGrayLevelEmphasis	MWT	0.02130	0.0004673	NO	µ_0_ < µ_1_	0.00088	0.00638
glszm_HighGrayLevelZoneEmphasis	MWT	0.03037	0.0004673	NO	µ_0_ ≥ µ_1_	2543	441
glszm_LargeAreaLowGrayLevelEmphasis	MWT	0.03403	0.0004673	NO	µ_0_ < µ_1_	0.012	0.095
glszm_LowGrayLevelZoneEmphasis	MWT	0.04484	0.0004673	NO	µ_0_ < µ_1_	0.0013	0.0108
glszm_SizeZoneNonUniformity	MWT	0.04484	0.0004673	NO	µ_0_ < µ_1_	2640	4581
glszm_SmallAreaHighGrayLevelEmphasis	MWT	0.03037	0.0004673	NO	µ_0_ ≥ µ_1_	1668	279
glszm_SmallAreaLowGrayLevelEmphasis	MWT	0.04732	0.0004673	NO	µ_0_ < µ_1_	0.0011	0.0087
ngtdm_Busyness	MWT	0.00487	0.0004673	NO	µ_0_ < µ_1_	0.45	2.48
ngtdm_Coarseness	MWT	0.04484	0.0004673	NO	µ_0_ ≥ µ_1_	0.00055	0.00031
ngtdm_Contrast	MWT	0.04990	0.0004673	NO	µ_0_ < µ_1_	0.092	0.127
ngtdm_Strength	MWT	0.01287	0.0004673	NO	µ_0_ ≥ µ_1_	1.38	0.33

MWT: Mann–Whitney test; TT: *t*-test; µ_0_: median group 0; µ_1_: median group 1; α_0:_ mean group 0; α_1_ mean group: SFs: significant features; SB: significative for Bonferroni.

**Table 3:** Demographic characteristics of MSS and MSI population.

MSI	TOTAL = 55	MSS	TOTAL = 60
Males	33	Males	30
Females	22	Females	30
Median age at diagnosis	64, 57	Median age at diagnosis	67, 46
Ascending colon	47	Ascending colon	46
Descending colon	8	Descending colon	14

**Table 4:** Summary of the relevant metrics from our study. AUC: Area Under the Curve CI: Confidence Interval. The Youden index was used to identify the optimal decision threshold that maximizes the sum of sensitivity and specificity. At the maximum Youden index, the corresponding specificity, sensitivity, accuracy, and precision were computed.

	AUC	95% CI (AUC)	SENSITIVITY	95% CI (SENSITIVITY)	SPECIFICITY	95% CI (SPECIFICITY)	ACCURACY	95% CI (ACCURACY)	PRECISION	95% CI (PRECISION)
MODEL I TEST	0.76	0.65–0.87 (DeLong)	0.72	0.58–0.85	0.75	0.61–0.88	0.71	0.61–0.8	0.71	0.57–0.84
MODEL I VALIDATION	0.74	0.56–0.92 (DeLong)	0.5	0.29–0.74	0.8	0.60–1.00	0.63	0.46–0.77	0.77	0.3–0.7
MODEL II TEST	0.85	0.73–0.96 (DeLong)	0.957	0.85–1.00	0.652	0.44–0.84	0.74	0.6–0.84	0.76	0.49–0.84
MODEL I VALIDATION	0.72	0.503–0.949 (DeLong)	0.73	0.50–0.94	0.44	0.13–0.78	0.63	0.43–0.79	0.69	0.48–0.89

## Data Availability

The raw data supporting the conclusions of this article will be made available by the authors on request.
